# Six-month multidisciplinary follow-up in multisystem inflammatory syndrome in children: An Italian single-center experience

**DOI:** 10.3389/fped.2022.1080654

**Published:** 2023-01-26

**Authors:** Gianvincenzo Zuccotti, Valeria Calcaterra, Savina Mannarino, Enza D’Auria, Stefania Maria Bova, Laura Fiori, Elvira Verduci, Alberto Milanese, Giuseppe Marano, Massimo Garbin, Salvatore Zirpoli, Valentina Fabiano, Patrizia Carlucci, Sara Olivotto, Laura Gianolio, Raffaella De Santis, Gloria Pelizzo, Elena Zoia, Dario Dilillo, Elia Mario Biganzoli

**Affiliations:** ^1^ Department of Biomedical and Clinical Science, University of Milan, Milan, Italy; ^2^ Pediatric Department, Buzzi Children’s Hospital, Milan, Italy; ^3^ Department of Internal Medicine, University of Pavia, Pavia, Italy; ^4^ Pediatric Cardiology Unit, Buzzi Children’s Hospital, Milan, Italy; ^5^ Pediatric Neurology Unit, Buzzi Children’s Hospital, Milan, Italy; ^6^ Department of Health Sciences, University of Milano, Milano, Italy; ^7^ Medical Statistics Unit, Department of Biomedical and Clinical Sciences, “Luigi Sacco” University Hospital, University of Milan, Milan, Italy; ^8^ Pediatric Radiology Unit, Buzzi Children’s Hospital, Milan, Italy; ^9^ Pediatric Surgery Department, Buzzi Children’s Hospital, Milan, Italy; ^10^ Intensive Care Unit, Buzzi Children’s Hospital, Milan, Italy

**Keywords:** multisystem inflammatory syndrome, children, SARS-CoV2, follow-up, COVID-19, complications

## Abstract

**Background:**

A severe multisystem inflammatory syndrome in children (MIS-C) related to SARS-CoV-2 has been described after infection. A limited number of reports have analyzed the long-term complications related to pro-inflammatory status in MIS-C. We evaluated multiorgan impairment at the 6-month follow-up in MIS-C.

**Methods:**

We enrolled 33 pediatric patients consecutively hospitalized for MIS-C and monitored for almost 6 months. The inter-relationship of patient's features and disease severity at admission with long term complications was studied by multivariate analysis.

**Results:**

Endo-metabolic derangement, cardiac injury, respiratory, renal and gastrointestinal manifestations and neurological involvement are part of the initial presentation. The most abnormalities appear to resolve within the first few weeks, without significant long term dysfunction at the 6-months follow-up, except for endocrine (non-thyroidal illness syndrome in 12.1%, insulin resistance in 21.2%) and neurological system (27.3% cognitive or psychological, behavioral, adaptive difficulties). Endocrine and heart involvement at admission represent a significant factor on the long term sequelae; however no association between severity score and long-term outcome was noted.

**Conclusions:**

The severity of initial clinical presentation may be associated to organ domain, however it is not related to long term sequelae. The prevalent organ restoration supports a predominant indirect immune-mediated injury triggered by a systemic inflammatory response; however a direct damage due to the viral entry could be not excluded. Eventhought our preliminary results seem to suggest that MIS-C is not a long-term risk condition for children health, a longer follow-up is mandatory to confirm this hypothesis.

## 1. Introduction

In March 2020, the World Health Organization declared COVID-19 a global pandemic (1). COVID-19 is a highly infectious disease responsible for significant rates of illness, hospitalization, and death among humans (1). The virus frequently causes respiratory manifestations such as fever, cough, and dyspnea. Gastrointestinal symptoms are also reported (2). In some cases, a severe infection can develop that can be fatal, manifesting as ARDS and multi-organ failure (2).

Although less frequently compared to the adult population, COVID-19 affects the pediatric age group. Recently, Ludvigsson et al. (2020) reported that children accounted for 1%–5% of COVID-19 cases, with a slight prevalence in the male sex (3). Most cases involve mild-to-moderate infection, 13% are asymptomatic, and 3% have severe disease (3); deaths were extremely rare (3).

However, the development of a severe multisystem inflammatory syndrome in children (MIS-C) related to SARS-CoV-2 has been described after infection (4–6).

Among the children with a confirmed exposure to COVID-19, less than 1% developed MIS-C (7). The demographic features of the patients revealed an age range between 7 and 10 years (8, 9), depending on the study, with a male predominance. The pathogenic mechanism of MIS-C remains unclear; the most accepted theory describes the primary role of a cytokine storm and the action of the adaptive immunity after SARS-CoV-2 infection (10, 11). Additionally, certain intrinsic susceptibility factors in the host have been described and the evidence of molecular mimicry was also presented for the disease pathogenesis (10).

As reported by Centers for Disease Control and Prevention (CDC), MIS-C may occur in subjects aged <21 years who test positive for current or recent SARS-CoV-2 infection by RT-PCR, serology, or antigen test or presented exposure to COVID-19 within four weeks prior to the onset of symptoms. Patients with MIS-C shows fever, biochemical evidence of inflammation and multisystem organ involvement (≥2, including cardiac, renal, respiratory, hematologic, gastrointestinal, dermatologic, or neurological system) requiring hospitalization and no alternative plausible diagnoses (12, 13).

The role of pro-inflammation after acute SARS-CoV-2 infection in poor prognosis and complications has been described in the literature (10, 14). A set of persistent symptoms occurring after documented SARS-CoV2 infection, defined as post-COVID syndrome (or long COVID), has been described in adults and the pediatric population (15), not only among those patients who were hospitalized with severe symptoms but also among those who were asymptomatic or with only mild symptoms. MIS-C may be recognized as post-COVID syndrome (16, 17); additionally, long-term complications related to pro-inflammatory status in children with MIS-C may not be excluded. To date, a limited number of reports have analyzed them (18–25).

The aim of this study was to evaluate multiorgan impairment at the 6-month multidisciplinary follow-up in children and adolescents with MIS-C, to assess whether MIS-C can be considered a long-term risk condition for child health; the interrelationship of patient's features and severity of disease at admission with long-term complications was also studied.

## 2. Patients and methods

### 2.1. Patients

In this prospective cohort study of patients, we included all children and adolescents consecutively hospitalized between November 2020 and March 2021 to the Pediatric Department of Children's Hospital Vittore Buzzi (Milan, Italy) for MIS-C, defined according to the Centers for Disease Control and Prevention (CDC) classification (12) and monitored for at least 6 months. As part of this study, for all patients, demographics and clinical characteristics, comorbidities, clinical presentation, multi-organ involvement, information about hospitalization [hospital stay length, need for pediatric intensive-care unit (PICU), ventilation support, outcomes], and laboratory data were recorded at admission. At the 6-month multidisciplinary follow-up, a clinical, biochemical, and instrumental assessment was repeated.

The study was conducted according to the guidelines of the Declaration of Helsinki and approved by the Institutional Review Board of the hospital (protocol number 2021/ST/138); the ethics committee approved the enrollment of all patients with a diagnosis of MIS-C admitted to the Vittore Buzzi Children's Hospital, Milan, from the beginning of the pandemic, including baseline and follow-up data. At the beginning of pandemic, MIS-C was not recognized as specific entity and the need of a long-follow-up were not clearly defined, thus no pre-existing ethical approval was available**.** After being informed about the nature of the study, children's guardians gave their written consent for the participation.

### 2.2. Methods

#### 2.2.1. Disease indicators and outcomes

The diagnostic procedure for confirming the MIS-C included all measures described according to the CDC case definition (12). As proof of SARS-CoV-2 infection we included positive reverse-transcriptase polymerase chain reaction or positive immunoglobulin M or G in a rapid test, or positive enzyme-linked immunosorbent assay, or antigen assay or known contact with a confirmed COVID-19 patient.

Severity of multisystemic involvement at admission was assessed devising a sub-score of severity for any of the domains involved: kidney, heart, gastrointestinal system, central nervous system, lung, skin-mucosal involvement, endocrine system, metabolic involvement, electrolyte imbalance.

Each sub-score ranges from 0 to 2 points and the total severity score was defined as the sum of each of the sub-scores (see following sections and Table 1 for details about the definition of each sub-score).

**Table 1:** Definitions of severity sub-scores and variables related to hospitalization.

Organ	SCORE		
	0	1	2
Kidney	Normal creatinine	Increase in creatinine <50% (26)	Increase in creatinine >50% (26)
Heart	Ejection fraction >45% (27)	Ejection fraction ≤45% (27)	Ejection fraction ≤35% (27)
Gastrointestinal system	None	Abdominal symptoms (28)	Abdominal symptoms and ultrasound pathological signs eco (28)
Central nervous system	None or aspecific neurological signs	mild encephalopathy + focal neurological signs +/− focal EEG abnormalities	encephalopathy + focal neurological signs + diffuse EEG abnormalities
Lung	None	respiratory symptoms and/or O2 therapy and/or Imaging alteration	Ventilation need
Fever	<3 days	3–7 days (29)	>7 days (29)
Skin/mucosal involvement	None	skin	Skin and mucosal
Endocrine system	None	<2 hormonal alterations (Including thyroid and/or adrenal hormones and/or insulin)	≥2 hormonal alterations
Metabolic involvement	None	<2 metabolic parameters (Including increased FBG, dyslipidemia, insulin resistance, hepatic alteration)	≥2 metabolic parameters
Duration of hospitalization in pediatric intensive care unit	None	≤3 days	>3 days
Hospitalization	<10 days	11–14 days	≥15 days
Weight loss	None	≤0.5 BMI-SD	>0.5 BMI-SD
Electrolyte imbalance	None	1 (sodium or potassium)	2 altered electrolytes

Furthermore, the outcomes considered in this work are referral to ICU during first hospitalization and long-term complications, the latter defined as presence at 6-months follow-up evaluation of at least one complication among the above domains plus presence of fever.

#### 2.2.2. Protocol therapy for MIS-C

As previously reported, to control and stop the hyperimmune response of MIS-C, a standard protocol for treatment based on a review of the current literature and mainly derived from Kawasaki disease experience and multidisciplinary expert consensus was adopted (30).

The cardinal treatment consisted of IVIG at 2 g/kg. In case of hemodynamic impairment, need for oxygen, and heart failure, corticosteroids (methylprednisolone) were added. In the case of mildly reduced LVEF or minimal oxygen support, we gave methylprednisolone 2 mg/kg for 5 days, followed by gradual tapering. If there was a significant oxygen requirement, mild organ injury, and/or moderately reduced LVEF, methylprednisolone 10 mg/kg for one day then 2 mg/kg for 5 days was given before tapering. If there was a need for respiratory support, inotropic support, moderate-to-severe organ damage, and/or severe heart dysfunction, and/or neurological involvement, we used a high methylprednisolone dose of up to 30 mg/kg for 3 days.

Supportive care (inotropes, fluid resuscitation, diuretics, oxygen and ventilation, antibiotics, anticoagulation, or anti-thrombotic prophylaxis) was considered in all the patients, according to clinical multidisciplinary team evaluation. As previously reported (30), the anti-thrombotic prophylaxis was started in all patients >12 years old, and was considered in patients <12 years old, if the D-dimer was high (>5 times the upper normal value) or if there was at least one known risk factors for thromboembolism. The anticoagulation therapy was prescribed in case of thrombosis and in case of severe LV dysfunction. With the reduction of the D-dimer or at the normalization of the LV function, heparin was shifted to low-dose aspirin for 3–4 weeks.

#### 2.2.3. Multidisciplinary evaluations

##### 2.2.3.1. Clinical evaluation

All child and adolescent physical examinations at the diagnosis and at the 6-month follow-up included anthropometric measurements of weight and height, body mass index (BMI) calculation, and evaluation of the pubertal stage as previously detailed (31).

Anthropometric evaluation: anthropometric measurements of weight and height, body mass index (BMI) calculation, and evaluation of the pubertal stage was made for each patient. The weight and height were measured using a mechanical column scale with an altimeter (Seca 711 and Seca 220), the arm and waist circumferences were measured with a tape measure (Seca 201), the tricipital skinfolds were measured using a caliper (Holtain 610). BMI (kg/m^2^), and the BMI Z-SCORE was established according to CDC growth chart reference values (32).

##### 2.2.3.2. Metabolic and hormonal evaluation

A blood sample was obtained in fasting state between 8:30 a.m. and 9:00 a.m. The metabolic profile including total and HDL cholesterol, fasting plasma glucose (FPG), and triglycerides (TG) was analyzed at the diagnosis and follow-up (33).

Within 24 h of admission, the evaluation of the endocrinological profile included: free T3 (FT3), free T4 (FT4) and TSH, fasting plasma insulin (FPI), cortisol, and ACTH levels.

The presence of non-thyroidal illness syndrome (NTIS) was considered as previously reported (33).

As a surrogate of insulin resistance (IR), two indexes were calculated as:

• Homeostasis model analysis—insulin resistance (HOMA-IR) index, defined as ((fasting plasma insulin (mU/L) × fasting plasma glucose (mg/dl))/405) (34). Pathological IR was defined as a HOMA-IR > 97.5th percentile for sex and pubertal stage (35).
• Triglyceride–glucose (TyG) index, calculated as (log(fasting triglycerides (mg/dl) × fasting plasma glucose (mg/dl)/2)) (36); the cutoff point for pathological IR was set at 7.88 (36–39).

Hormonal panels were repeated at the 6-month follow-up.

##### 2.2.3.3. Cardiologic assessment

For all the patients, at admission (and during the hospitalization if necessary), we considered echocardiographic ventricular measurements and cardiac electric abnormalities at the ECG and arrhythmias.

Left ventricular (LV) function was qualitatively and quantitatively assessed by Philips Affinity 70 or Vivid S5 GE Healthcare, and the left ventricular ejection fraction (LVEF) was calculated according to Simpson's biplane method (30).

To decide the therapeutic dosage of steroids, left cardiac function was classified as follows: normal (LVEF > 55%), mildly–moderately reduced (LVEF ≤45%), or severely reduced (LVEF ≤35%). We also measured mitral regurgitation (qualitative and semi-quantitative evaluation), the degree of pericardial effusion (mild if ≤5 mm, moderate if >5 mm), and the coronary artery size (Montreal Z-score) (30).

In additional to the conventional echocardiography at six months after discharge, an echocardiographic (by E95 GE Healthcare) strain analysis (Global Longitudinal Strain of left ventricle (GLS); Left Atrial Reservoir Strain (AS); Right Ventricle Free Wall Strain (RVS)) was performed to detect any subclinical cardiac abnormalities. Off-line analysis was performed offline with independent software (TOMTEC Imaging Systems GmbH, www.tomtec.de/technical-docs). Based on the echocardiographic speckle-tracking algorithm in each subject, apical four-chamber, two-chamber, and three-chamber views were analyzed for GLS; the apical four-chamber view was analyzed for AS; and a dedicated apical projection for the right ventricle was analyzed for RVS. Diastolic function [early and late mitral inflow peak velocities, tissue doppler images (TDI) of septal and lateral annular peaks] was also evaluated. Furthermore, all patients underwent a Holter recording.

##### 2.2.3.4. Lung assessment

Imaging was obtained by x-ray and lung ultrasonography (US). A chest x-ray was performed at hospital admission, generally within 24 h of admission, and lung US was performed after IVIG therapy. At six months after discharge, US was performed.

For the x-ray, the following features were considered: the presence of pulmonary opacities, presence and degree (mild/moderate) of peri bronchial thickening, presence of pleural effusion, and cardiomegaly.

A severity score of pulmonary involvement was calculated adopting a simplified version of the Radiographic Assessment of Lung Edema Score, previously used both in adults (40) and children MIS-C patients (41). A score of 0–4 was assigned to each lung depending on the extent of involvement (0 = no involvement; 1 = <25%; 2 = 25%–50%; 3 = 50%–75%; 4 = >75% involvement), with a final severity score ranging from 0 to 8.

At lung US, each lung was divided into 6 regions, for a total of 12, considering anterior, lateral, and posterior regions of both upper and lower parts of the chest, in accordance with Copetti and Cattarossi (42). A US severity score of pulmonary involvement was calculated using the global lung US aeration score, which considers four validated US patterns: normal, characterized by the presence of lung sliding and A-lines (0 points); presence of multiple well-defined vertical B-lines extending from the pleural line or a small subpleural consolidation (1 point); presence of multiple confluent vertical B-lines extending from the pleural line or a small subpleural consolidation (2 points); presence of lung consolidation with air bronchograms (3 points). The total score is obtained by summing the individual scores of all the regions, ranging from 0 to 36 (43).

##### 2.2.3.5. Neurological assessment

All the patients were assessed at admission and during the hospital stay in accordance with our previously reported assessment protocol (44).

Neurological examination was carried out by a child neurologist, and neurological signs and symptoms were classified as:

- Signs of altered mental status—encephalopathy (altered state of consciousness, irritability or agitation, behavioral changes, i.e., emotional lability/impulsivity, mood deflection/anxiety);
- Neurological signs (abnormal eye movements, cranial nerve dysfunctions, gait disturbances, hemiparesis/hemiplegia, flaccid paralysis, movement disorders, speech disorders, memory deficits, visual/auditory hallucinations, seizures, muscle tone alterations);
- Non-specific symptoms (apathy, lack of appetite, asthenia, changes in the sleep/wake rhythm, headache, limb or trunk pain, paresthesia/anesthesia).

EEG was performed with wake and sleep recording in subjects with altered mental status. EEG abnormalities were classified as focal or diffuse according to the characteristics of the background activity slowing and the presence and localization of epileptiform changes and periodic and rhythmic patterns.

Brain MRI and CSF sampling (including SARS-CoV-2 PCR and neurotropic viral PCR, isoelectrofocusing and CSF IgG, IgM and light chains) were performed in subjects with EEG abnormalities.

A severity score was assigned on the basis of the number and type of clinical and instrumental findings:

SCORE 2—*encephalitis* (encephalopathy + neurological signs + diffuse EEG abnormalities).
SCORE 1—*mild clinical involvement* (mild signs of altered mental status + one or two focal neurological signs +/− focal EEG abnormalities).
SCORE 0—*aspecific signs and symptoms* (no neurological signs, or with one focal or aspecific neurological signs).

Six months after discharge, neurological assessment, carried out by a child neurologist, included:

- Neurological examination including non-verbal IQ and *ad hoc* structured interview focusing on school performances, memory, and attention);
- Anamnesis focusing on daily life habits, school performances, social skills, behavior, anxiety, depression with an *ad hoc* structured interview;
- Structured interview evaluating behavioral and adaptive functioning—Child Behavior Checklist parent's questionnaire (CBCL);
- Structured interview evaluating quality of life (QoL)—The PedsQL—Pediatric Quality of Life Inventory™_)_;
- EEG—asleep and awake—in subjects with previous encephalitis.

At this time, a severity score was assigned as follow:

SCORE 2—neurological/neuropsychological signs or EEG abnormalities AND psychological difficulties or behavioral and adaptive problems or low QoL;
SCORE 1—neurological/neuropsychological signs or EEG abnormalities OR psychological difficulties or behavioral and adaptive problems or low QoL.

##### 2.2.3.6. Gastroenterological evaluation

Abdominal signs (diarrhea, vomiting, or both) were recorded. An abdominal ultrasound was performed at the diagnosis in all the patients; ultrasound abnormalities of the appendix and gallbladder (increased wall thickening and/or stones) and increased ileal and/or cecal wall thickening were considered.

US abdomen was repeated at the follow-up when abdominal symptoms were evaluated.

#### 2.2.4. Discharge criteria

All patients were discharged upon achievement of the following conditions:

• Good general health condition
• Asymptomatic
• Afebrile ≥ 72 h
• Decreased inflammatory markers
• Suspension of supportive care
• Decreased doses of corticosteroid therapy

**Figure 1:**
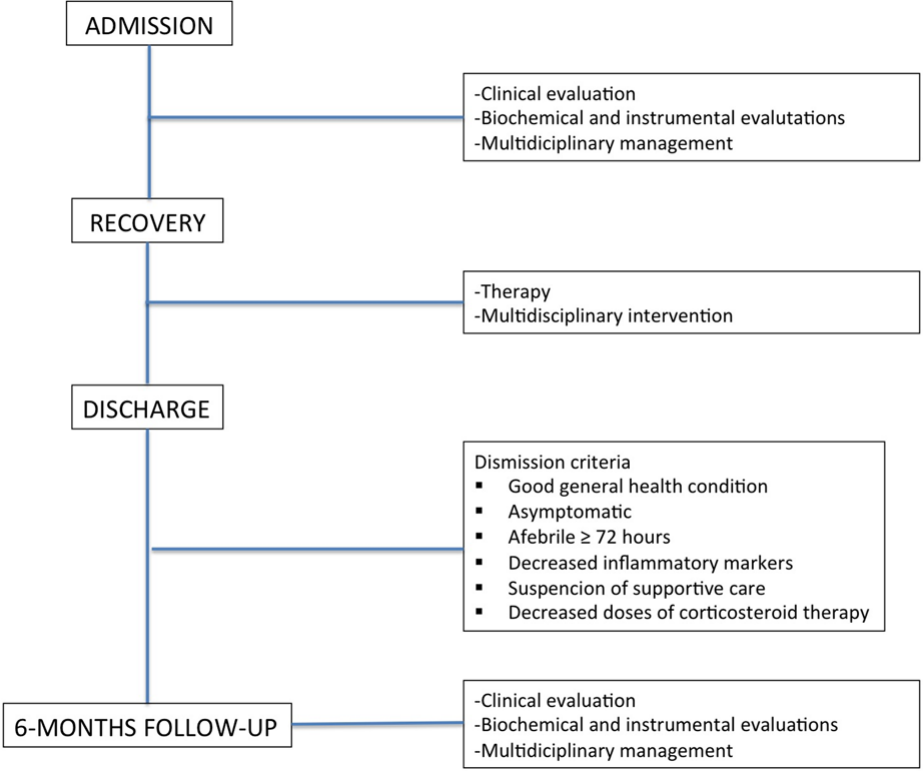
From admission to follow-up flow chart.

In Figure 1, from a flowchart from admission to follow-up is reported.

### 2.3. Statistical methods

A descriptive analysis was performed for all the demographic, laboratory, and clinical variables both at admission and at the 6-month follow-up visit on the 33 subjects recruited for the study: since the non-symmetrical distribution of the variables, continuous variables were summarized using the median and quartiles; categorical variables were reported using absolute frequencies and percentages.

With the aim of characterizing multiorgan impairment of the patients, a multiple correspondence analysis (MCA) (45) was performed. MCA is a multivariate statistical method of analysis, which allows to identify association patterns among the categorical variables and to describe profiles of individuals. This is achieved through projections of variable categories and individuals in a low-dimensional space (usually two/three dimensions) that preserves the largest amount of the total variability of the data. Results of MCA analysis, i.e., the above projections, are graphically represented in scatterplots (“MCA scatterplots”). The proportion of data variability preserved in the MCA scatterplot is measured by the index called “explained inertia”. It is worth noting that the explained inertia is also a measure of association: values of explained inertia close to 100% indicate that all the variables included in the analysis are strongly associated one another, while values close to 0% indicate a situation near to the independence between each couple of variables.

In the present work MCA was carried out on the sub-scores of organ and system severity, and, in order to investigate the association between patterns of multiorgan impairment and hospitalization-related variables, on the following variables: fever duration, length of hospitalization, weight loss (see Table 1 for definitions). Three subjects out of the total 33 were excluded because of presenting a large proportion of missing data. The association patterns were evaluated by examining the position of variable's categories in the MCA scatterplot: points close to one another indicate categories that tend to be co-present in subjects. In a similar fashion, patient profiles were evaluated by examining the position of points in the respective scatterplot, see the previously mentioned textbook (45) for details.

With the aim of evaluating the association of patient severity at admission with referral of the patient to ICU during first hospitalization and long-term complications, logistic regression was performed for 23 patients with complete data records. In the logistic models, referral at ICU and long-term complications were considered as response variables, while the severity score at admission was included as explanatory variable. Furthermore, sex and age, considered as potential confounding variables, were also included in each model (as explanatory variables). Results were reported in terms of adjusted ORs with respective 95% C.I.s, and Wald tests on regression coefficients (Student t distribution). All tests were two-tailed, and the level of significance was set conventionally at *α* = 0.05.

Statistical analyses were conducted using the software R (R Core Team, 2021; R version 4.1.2) (46) with the package FactoMineR added (47).

## 3. Results

In Tables 2, 3, the severity scores according to system involvement and the values of biochemical parameters at admission and at the 6-month follow-up are reported.

**Table 2:** Frequency distributions at admission and 6-month follow-up for severity sub-scores and variables related to hospitalization.

Organs	Score	Admission/Hospitalitation N (%)	6-month follow-up N (%)
Kidney	0	16 (48.5)	33 (100)
	1	6 (18.2)	0 (0)
	2	4 (12.1)	0 (0)
	NA	7 (21.2)	0 (0)
Heart	0	21 (63.6)	33 (100)
	1	7 (21.2)	0 (0)
	2	5 (15.2)	0 (0)
Gastrointestinal system	0	0 (0)	33 (100)
	1	3 (9.1)	0 (0)
	2	30 (90.9)	0 (0)
Central nervous system	0	14 (42.4)	14 (42.4)
	1	18 (54.5)	10 (30.3)
	2	1 (3.0)	9 (27.3)
Lung	0	8 (24.2)	26 (79)
	1	13 (39.4)	7 (21)
	2	12 (36.4)	0 (0)
Fever	0	0 (0)	33 (100)
	1	27 (81.8)	0 (0)
	2	6 (18.2)	0 (0)
Skin/mucosal involvement	0	16 (48.5)	33 (100)
	1	5 (15.2)	0 (0)
	2	12 (36.4)	0 (0)
Endocrine system	0	3 (9.1)	29 (87.9)
	1	10 (30.3)	4 (12.1)
	2	17 (51.5)	0 (0)
	NA	3 (9.1)	0 (0)
Metabolic involvement	0	6 (18.2)	26 (78.8)
	1	13 (39.4)	7 (21.2)
	2	11 (33.3)	0 (0)
	NA	3 (9.1)	0 (0)
Duration of hospitalization in pediatric intensive care unit	0	11 (33.3)	Not applicable
	1	12 (36.4)	
	2	10 (30.3)	
Hospitalization	0	2 (6.1)	Not applicable
	1	21 (63.6)	
	2	10 (30.3)	
Weight loss	0	7 (21.2)	33 (100)
	1	25 (75.8)	0 (0)
	2	1 (3.0)	0 (0)
Electrolyte imbalance	0	5 (15.2)	33 (100)
	1	15 (45.5)	0 (0)
	2	9 (27.3)	0 (0)
	NA	4 (12.1)	0 (0)

*N* = absolute frequency; % = percentage.

**Table 3:** Biochemical parameters at admission and 6-months follow-up. For each variable, reported in the table there are median values, 25-th and 75-th percentiles of values recorded at admission and 6 months follow-up.

Variables	Admission	6-month follow-up
Fasting blood glucose (nv < 100)	110.50 (90.50, 123.75)	87.00 (83.00, 91.00)
Tryglicerides (nv < 150 mg/dl)	195.00 (126.00, 319.00)	78.00 (54.00, 97.00)
Tryg-index (nv <7.88)	9.22 (8.87, 9.64)	8.11 (7.78, 8.31)
HOMA-IR	4.95 (3.78, 9.44)	2.06 (1.28, 3.17)
Total cholesterol (vn <190 mg/dl)	120.00 (87.50, 164.00)	156.00 (143.00, 168.00)
HDL cholesterol (vn. > 40 mg/dl)	17.00 (8.00, 24.00)	47.00 (43.00, 53.00)
GB (n°/mm3) nv < 2 years: 6–17; 2–8 years: 5.5–15.5; 8–16 years: 4.5–13.5; >16 years: 4.5–13	9,370.00 (6,680.00, 13,655.00)	6,470.00 (5,735.00, 7,047.50)
Platelets (nv 150,000-500,000 mm^3^)	161,000.00 (108,000.00, 215,000.00)	290,000.00 (260,750.00, 356,500.00)
Hb (g/dl) nv < 2 years > 105; 2–12 years > 115; 12–18 years males > 130; 12–18 years females > 120	10.80 (10.20, 11.90)	13.15 (12.62, 14.15)
creatinin.(mg/dl) Nv <1.yr 0,15-0,4; .1–3 yrs 0.2–0.35;.3–7 yrs; 0.25–0.45;.7–11 yrs 0.35–0.6;.11–18 yrs 0.45–0.75	0.54 (0.42, 0.74)	0.46 (0.37, 0.55)
C Reactive Protein (nv <10 mg/L)^a^	199.90 (103.80, 277.70)	1.80 (1.10, 4.15)
NT-proBNP (nv <125 ng/L)	10,225.00 (1,256.00, 14,825.00)	Not defined
Troponin (nv <125 ng/L)	34.00 (13.25, 72.50)	5.00 (4.00, 5.00)
Fibrinogen^b^ (nv <4 g/L)		
Normal	1 (3.0)	19 (57.6)
Pathologic	25 (75.8)	14 (42.4)
NA	7 (21.2)	0 (0.0)
Total protein (nv >6.5 g/dl)	5.80 (5.30, 6.50)	Not defined
Albumin (nv >3 g/dl)	2.70 (2.40, 2.88)	4.40 (4.30, 4.70)
D-dimer (nv <200 μg/L)	2,756.00 (2,107.00, 4,581.00)	323.00 (237.00, 531.00)
IL6 (nv <7 ng/L)	162.50 (95.50, 481.00)	
Sodium (nv 135–145 mEq/L)	132.00 (130.00, 136.00)	140.00 (139.00, 141.00)
Potassium (nv 3.5–5 mEq/L)	3.50 (3.00, 3.90)	4.00 (3.90, 4.20)
Ferritin (nv 7–140 μg/L)	742.00 (380.50, 1,230.75)	30.00 (26.00, 41.00)
FT3 (nv 3.5–6.3 pmol)	2.70 (2.02, 3.75)	5.90 (5.20, 6.30)
FT4 (nv 9–19.3 pmol/L)	12.20 (11.03, 14.07)	12.00 (11.30, 12.50)
TSH (nv 0.5–4.2 µIU/ml)	2.27 (1.18, 3.08)	2.15 (1.66, 2.76)
ACTH (vn. 5–60 pg/ml)	12.00 (4.00, 17.00)	24.00 (18.85, 32.00)
Cortisol (vn. 48–195 ng/ml)	122.00 (53.50, 198.25)	89.50 (76.75, 121.25)

nv, normal values.

* C Reactive Protein at time 6 calculated on subjects with values higher than the detection threshold of the instrument. The C Reactive Protein values at time 6 were below the detection limits of the instrument for 17/33 subjects (51.5%).

^b^ Fibrinogen was classified as normal and pathologic according to the cut-off of 4 g/L.

### 3.1. General clinical conditions

We considered 33 children and adolescents (25M/8F, 51.5% prepubertal, 75.7% Caucasian) with a median age of 10.00 (7.00, 14.00) years. No underlying diseases and/or previously known comorbidity were recorded in the patients. All subjects were unvaccinated for SarsCov2.

The clinical severity and biochemical features of the patient at admission and follow-up are reported in Tables 2, 3.

Median value of the duration of fever were 6.00 days.

Hospitalizations in a pediatric intensive-care unit were recorded in 22/33 of cases (66.6%) with a median PICU stay of 3.00 days. Mechanical ventilation was required in 12/22 (54.5%) subjects.

Overall, a median hospitalization duration of 12.00 days was recorded.

Weight loss was reported in 26/33 (78.8%) of children.

No patients died. All patients achieved a full clinical recovery.

At the 6-month follow-up all patients showed good general clinical conditions with weight restoration in all.

### 3.2. Skin and/or mucosal involvement

As reported in Tables 2, 3, skin and/or mucosal involvement were detected in 17/33 of patients (51.8%). In particular, cutaneous involvement, conjunctivitis, and mucosal involvement were found in 72.7%, 60.6%, and 27.3%, respectively. Cervical lymphadenopathy was also recorded in 18.2% of the patients.

At the 6-month follow-up, no patient with skin and/or mucosal involvement was detected (see Table 2).

### 3.3. Metabolic and endocrine system

At admission, at least two pathological metabolic parameters were observed in 24/33 (72.7%) of patients (see Table 2). In particular, increased fasting blood glucose levels were detected in 21/33 (63.6%), low-HDL in 29/33 (87.9%), and hypertriglyceridemia in 17/33 (51.5%).

Pathological TyG index was detected in 32 subjects (97.0%). It was possible to calculate the HOMA-IR index for only 19 patients, and among these, 18 (54.5% of total and 94.7% of measured) were insulin resistant.

A total of 27 of the 33 (81.8%) children showed endocrinological derangement, including NTIS in 29 of patients (87.8%; in particular, only low FT3 level in 17/29 cases, low FT3 associated with combinations of FT4 and/or TSH abnormalities in 9/29 and isolated high TSH level in 3/29 subjects), pathological cortisol and/or ACTH levels in 15 subjects (45.4%).

At the 6-month follow-up, all metabolic and endocrinological data were recorded. Insulin resistance persisted in seven (21.2%) patients and NTIS in four children (12.1%; pathological level of FT3 and TSH in two and two subjects, respectively), with resolution of the other metabolic and endocrinological parameters (see Table 2).

### 3.4. Heart

The median value of EF% was 50.00. EF impairment was detected in 46.15% of subjects. Valvular regurgitation, coronary artery dilation (Boston *Z* score < 2.5), and pericardial effusion were also detected in 72.8%, 3%, and 21.3% respectively.

Electrocardiogram anomalies were found in 14 patients (42.4%), without a difference between different EF groups. In particular, as previously reported (30), we detected:

- ST segment changes and/or negative T waves (38%); type-1 Brugada pattern (9%) and atrial fibrillation (3%);
- rhythm anomalies including first-degree heart block, escape junctional rhythm alternating with sinus rhythm with premature ventricular contractions, and a short coupling interval (9% of patients);
- atrial fibrillation in one patient.

At the 6-month follow-up, resolutions to all the cardiac complications were obtained, including right and left ventricular strain values and diastolic function (RV systolic function: TAPSE 20.7 (±1.9) mm, s wave 0.14 (±0.02) m/s, RVFWLS (%)—28.1 (±3.9). LV systolic function: EF 63.3% (±4.5), LV GLS (%)—22.1 (±1.9). LV diastolic function: E/A 1.7 (±0.4), E/e'5.8 (±0.9), LAS (%) 45.0 (±6.8).

On Holter examination, no repolarization or rhythm alterations were detected in all patients, except one, who developed Brugada phenocopy during the subacute phase, in the presence of negative family and medical histories. Brugada syndrome was also suspected at Holter ECG monitoring at 6-month follow-up and the diagnosis was confirmed eight months later, by a provocative test with ajmaline (48).

The only patient with coronary artery dilatation normalized, and no new alterations appeared in any patients.

### 3.5. Central nervous system

At admission, neurological involvement was documented in 30/33 (90.9%) patients. Signs of altered mental status of variable severity were present in 14/33 patients (42.42%). Focal neurological signs were observed in 10/33 patients (30.30%), and non-specific symptoms were reported in 24/33 patients (72.73%).

EEG was performed in 10 children. Abnormalities were detected in 7/10. In the child with severe encephalopathy, EEG was dominated by diffuse high-amplitude delta slow activity. Focal EEG abnormalities, predominant over the posterior regions, were detected in the other six children. Five patients underwent brain MRI, with normal findings. CSF sampling was performed in five patients. All the examinations were normal. SARS-CoV-2 was not detected.

One child (3%) presented encephalitis with severe encephalopathy, focal neurological signs, and diffuse EEG abnormalities (severity SCORE: 2). Eighteen children (39.4%) had mild clinical involvement with signs of altered mental status and focal neurological signs with or without focal EEG abnormalities (severity SCORE 1). Eleven children (33.3%) had only focal or nonspecific neurological signs and symptoms; among them, four were completely asymptomatic (severity SCORE 0).

At the 6-month follow-up, the neurological examination and nonverbal intelligence were normal in all children. Seven children (21.21%) reported worsening of school performance, memory, and attention difficulties. Seventeen children (51.51%) reported psychological symptoms in variable combinations (socialization difficulties, behavior and anxiety or somatization disorder) that were confirmed by CBCL results in four. Low QoL was reported by five children (15.15%). Focal EEG abnormalities were documented in two children (the one with previous encephalitis and one with previous focal EEG abnormalities).

Nine children (27.3%) presented neurological signs or EEG abnormalities AND psychological, behavioral, or adaptive difficulties or low QoL (SCORE 2) and ten children (30.3%) presented either neurological signs or EEG abnormalities OR psychological, behavioral, or adaptive difficulties or low QoL (SCORE 1).

### 3.6. Lung

Overall, 25 out of 33 (75.8%) subjects showed lung involvement (see Table 2).

A total of 12 out of 33 (36.4%) patients required non-invasive ventilation (severity score 2), and 13 (39.4%) were symptomatic and/or showed imaging alterations and/or needed oxygen therapy but did not require ventilatory support (severity score 1). Patients who satisfied criteria for severity score 2 were not counted for severity score 1.

A lung US and chest radiograph were performed in 30 patients at admission (90.9%).

US abnormalities were found in 23 out of 30 (76.6%), with a score ranging from 2 to 8 points.

Radiographic abnormalities were found in 25 (83.3%), with a score ranging from 2 to 6 points out of 8; only four patients (16%) with mild x-ray involvement (score 2/8) had no US pathologic findings. Perihilar interstitial thickening was the most common finding, present in all pathologic chest x-rays.

Among patients with US pulmonary involvement, only two patients (8.7%) had a normal chest x-ray; seven patients (30.4%) showed mild radiographic findings with a total score of 1–2; more severe radiographic involvement was observed in 10 (43.5%) and 4 (17.4%) who had a US scores of 3–4 and 5–6, respectively; no patient had an RX score > 6.

The median chest radiograph severity score was 2 (IQR 2–4) and the median US score was 3 (1.5–6).

At the 6-month follow-up, 7 (21%) out of 33 subjects reported shortness of breath during exercise, while no one showed imaging alterations.

### 3.7. Gastrointestinal system

At admission, at least one abdominal symptom and/or ultrasound sign was detected in all patients. In particular, vomiting and abdominal pain were reported in 60.6% and 57.6% of patients, respectively. In more than 90% of children, ultrasound abnormalities were found, including US abnormalities of the appendix (3%), increased gallbladder wall thickening (21.1%), and increased ileal and/or cecal wall thickening (42.4%).

No gastrointestinal symptoms were recorded at the 6-month follow-up.

### 3.8. Kidney

At admission, kidney functional injury was noted in 10/33 (30.3%) of patients, without residual signs at the 6-month follow-up.

### 3.9. Multivariate analysis

#### 3.9.1. Patterns of multiorgan impairment

**Figure 2:**
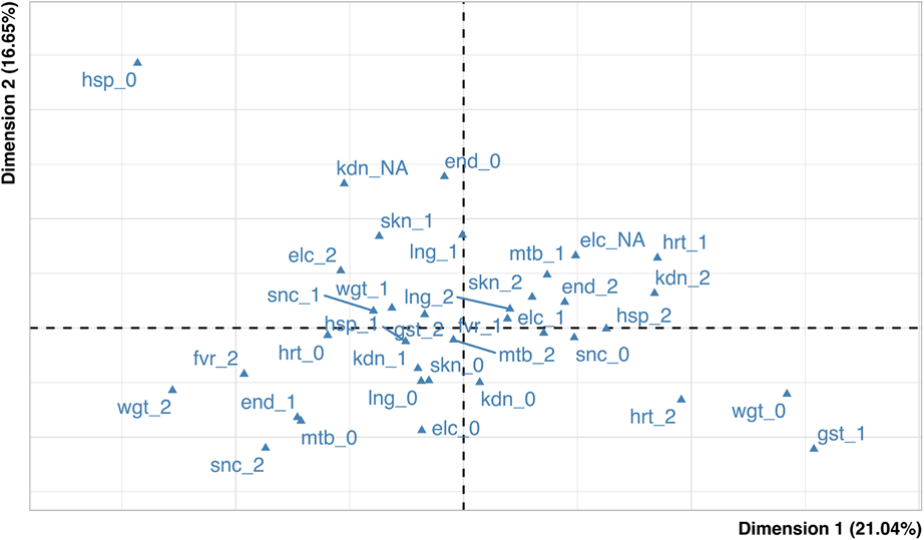
Map of variables. In the figure the blue triangles represent the active variables. kdn: kidney, hrt: heart, gst: gastrointestinal, snc: central nervous system, lng: lung, fvr: fever duration, skn: skin-mucosal involvement, end: endocrine system, mtb: metabolic involvement, hsp: duration of hospitalization, wgt: weight loss, elc: electrolyte imbalance.

For exploratory purposes, the association between the variables in Table 1 was evaluated by MCA methods. The percentage of explained inertia for the MCA scatterplot (Figure 2) is 37.6%, indicating a low amount of association among the variables.

After a close inspection (performed using indices of quality of representation) (45), the modalities showing relevant association were related to the “heart” and “endocrinology” domains: in Figure 2 it may be seen that the ​​categories heart severity = 1, heart severity = 2 and endocrine severity = 2 are relatively close one another (on the right side of the graph), and the same holds true for the categories heart severity = 0 and endocrine severity = 1 (on the left side on the graph). The presence of the above two groups of categories suggests that severe conditions of heart tend to “come together” with severe conditions of endocrine system, while healthy conditions of heart associate with mild severity of endocrine system.

**Figure 3:**
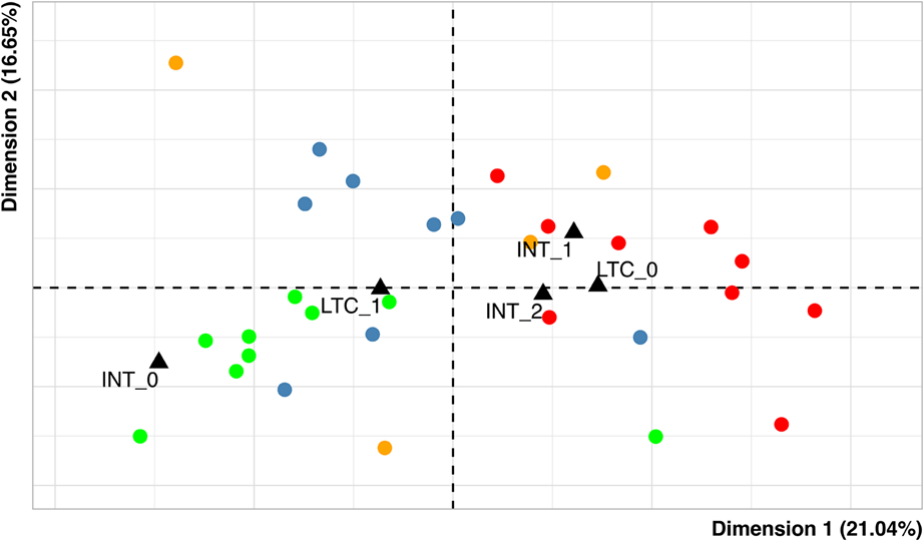
Map of individuals. Each dot represents a single patient and the variables duration of hospitalization in pediatric intensive care (INT) and the presence of long-term complications (LTC) are used as supplementary variables (black triangles). - green: patients with heart severity = 0 and endocrine severity = 1; - red: patients with heart severity = 1,2 and endocrine severity = 2; - blue: patients with heart severity = 0 and endocrine severity = 2; - orange: others.

Figure 3 shows the representation of individuals. Distinct colors were used according to the effective scores of severity of heart and endocrine involvement items, for a total of four distinct sub-groups. The main sub-groups consist of:

- patients with no heart involvement and moderate endocrine involvement (green; nine subjects);
- patients with no heart involvement and severe endocrine involvement (blue; eight subjects);
- patients with moderate/severe heart involvement and severe endocrine involvement (red; nine subjects).

The fourth group (orange) in the graph gathers a small group of patients (*n* = 4) presenting other combinations of the “heart” and “endocrinology” modalities.

In addition, to evaluate whether the profiles above described could have distinct outcome prevalence, we considered the relationships of profiles with length of ICU hospitalization and long-term complications. These variables were represented in Figure 3 as supplementary variables (this technique allows to evaluate further relationships without affecting the patterns of the points in the MCA scatterplots) (45). It emerged that:

(1) Brief and long permanence categories (respectively, less and more than three days) in the ICU are represented on the right side of the plot, while no admission is on the left side. These positions suggest that patients with a higher level of heart and endocrine involvement (red) have a higher prevalence of ICU admission, irrespective of the duration (8/9 patients, 88.9%) while less severe patients (green) have a lower one (5/9 patients, 55.6%). The patients with no heart involvement and severe endocrine involvement (blue) also have a high prevalence of ICU stay (6/8 patients, 75.0%), but the length of stay could be shorter with respect to the more severe ones; in fact, the length of stay is higher than three days for 4/9 patient in the red group, and for only 1/8 patients in the blue one.
(2) The presence of long-term complications is represented on the left side of the plot, while their absence is on the right side. These positions suggest that patients with a lower level of heart and endocrine involvement (green) have a higher prevalence of complications at follow-up (7/9, 77.8%), while more severe patients (red) have a lower one (4/9 patients, 44.4%). The patients with no heart involvement and severe endocrine involvement (blue) also have a high prevalence of long-term complications: 6/8 patients (75.0%).

#### 3.9.2. Admission to ICU, long-term complications, and their association with severity of disease

Over the total 33 patients, 22 (66.7%) were admitted to intensive care; long-term complications were present in 23 patients (69.7%). Of the 22 patients admitted to ICU, 13 (59.1%) had long-term complications, while this proportion was higher in those not admitted to ICU (10/11 patients, 90.9%). However, no statistically significant difference emerged by a formal comparison: this was assessed by the Fisher exact test, obtaining an OR of long-term complications of 0.15 (patients admitted to ICU vs. patients not admitted), 95% C.I.: 0.003 to 1.430 (*p* = 0.1085). Concerning the types of complication, the most widespread were neurological and metabolic (second column of Table 2); in particular, neurological ones were present in almost the totality of patients with complications at follow-up: 19/23 (82.6%).

Moreover, if we consider only the neurological domain, it can be noted in Table 2 that there is a relevant number of patients (*n* = 9) with severe long-term complications (i.e., severity = 2), which is in contrast with the situation at admission, where only one patient resulted with severe neurological impairment. By making further tabulations, among the 9 patients with severity = 2 at follow-up, 7/9 had severity = 1 for the neurological domain at admission, and 2/9 had severity = 0 at admission.

However, it should be noted that the neurological scores were defined differently at admission and follow-up times, respectively (see 2.2.3 Multidisciplinary evaluations: Neurological assessment).

The association between the intensive-care variable (dichotomized as access/non-access to intensive care) and the severity score was investigated through a logistic regression analysis. The adjusted OR was 1.71 (95% CI: 0.92–3.17, *p*-value = 0.088). With limited statistical evidence, the result can be interpreted as a moderate association between the two variables.

Finally, the association between the long-term outcome variable and the total score was studied through logistic regression analysis as before. The adjusted OR was 0.834 (95% CI: 0.55–1.27), without statistical evidence to conclude for the presence of association (*p* = 0.400).

## 4. Discussion

MIS-C is a severe consequence of SARS-CoV2 infection in children that is associated with significant multiorgan compromission (49). Literature data on the long-term outcome of MIS-C is limited (18–25).

We reported the evolution of the multiorgan impairment at the 6-month multidisciplinary follow-up, showing that despite the severity of initial presentation, most abnormalities appear to resolve within the first few weeks, without significant long-term organ dysfunction, except for the endocrine and neurological systems (4). Endocrine and heart involvement at admission represent a significant factor on the long-term sequelae; however, no association between the severity score and long-term outcome was noted.

Additionally, for cardiac injury, respiratory and gastrointestinal manifestations, renal dysfunction, and neurological involvement that are usually described in MIS-C, in our cohort, at admission, most of the children showed endocrinological derangement including NTIS, impaired glucose–insulin metabolism, and pathological cortisol and/or ACTH levels.

NTIS can be detected in many critical illnesses and represents imbalances in hormonal production, metabolism, and action (50). This condition was also frequently observed during the COVID-19 pandemic (51–55) and has been correlated with the severity of COVID-19 disease (56).

The thyroid is a crucial organ in maintaining many long-term functions of the body, in particular, cardiovascular, respiratory, and catabolic tasks (33). The alteration of thyroid function in the acute phase, typically associated with low levels of total triiodothyronine (T_3_), may support the predominance of catabolic situation during hyper-inflammatory condition and consequent adaptive metabolic response to save energy. Similarly to other reports (57, 58), being referral center for MIS-C, we reported an high percentage of patient (66%) with severe disease requiring admission to ICU. The link between endocrinological derangement and the severity score at admission may support that the cytokines, released during illness, represent a major determinant of NTIS (59); however, the exact mechanism through which the hormone profile influence the clinical course, as well as that of many other critical illnesses, is not clear yet.

The adaptive response allows vital organs to conserve energy, and it appears to be driven by counter-regulatory hormones and cytokines, which may also be mediators of IR, and result in mild/moderate hyperglycemia that provides fuel for the brain after stress conditions. In this pediatric cohort, the copresence of alteration of IR markers and lipids, thyroid values, and electrolytes confirm catabolic illness and the impairment of glucose homeostasis within the body. As previously discussed, the pathological values in HOMA-IR and TyG indexes suggest a both hepatic and peripheral impaired insulin action (36). The action of counter-regulatory hormones on IR in skeletal muscles might be mediated through an increase in the circulating free fatty acid level, despite hyperinsulinemia (60, 61).

Our results are not surprising in terms of adaptive metabolic response. Nevertheless, in this clinical context and also considering the persistence of long-term alterations on thyroid and glucose–insulin metabolism, a bidirectional relationship between COVID-19 and neuroendocrine system could be also considered.

The high ACE2 and TMPRSS2 expression in the thyroid may facilitate Sars-Cov2 entry. Additionally, as reported by Scappaticcio et al. (62), there is strong evidence that the entire hypothalamic–pituitary–thyroid (HPT) axis may be an important target for SARS-CoV-2 damage; selective transient pituitary dysregulation due to both the direct cytotoxic effect of the virus at the pituitary level and an indirect effect *via* the activation of proinflammatory cytokines may be considered. This COVID-19-induced damage to the hypothalamus and pituitary was also supported by electrolyte abnormalities reported at admission that may be associated with a syndrome of inappropriate antidiuretic hormone resulting in hyponatremia (63, 64).

A relationship between COVID-19 and pancreatic function has been also described. The pancreatic *β* cells are permissive to SARS-CoV-2 infection with receptor angiotensin-converting enzyme 2 (ACE2) as its entry, promoting cell damage and functional impairment.

The response of the hypothalamic–pituitary–adrenal axis in critically ill patients is poorly understood; however, an appropriate activation of HPA is described (65, 66). In children, altered cortisol levels may occur; high cortisol levels likely reflect more severe stress, whereas low levels may point to an insufficient response to stress, labeled relative adrenal insufficiency. ACTH mediate cortisol release in the acute phase (67), whereas an “inadequate” response to ACTH is involved during prolonged critical illness.

Considering the ACE2 and TMPRSS2 expression in adrenocortical cells, a direct virus–cell interaction may be not excluded. Additionally, the role of several cytokines on modulation cortisol production as well as the glucocorticoid receptor number and/or affinity should be considered. The hyper-inflammatory state and cytokine storm in MISC could be crucial in the appropriate activation of HPA, also considering the spontaneous recovery of the alterations during long-term follow-up.

Endocrine and heart involvement seem to be correlated, and their association represent a significant factor on the long-term sequelae.

As also previously reported (30), in the acute phase, the cardiovascular system is generally affected with mild-to-moderate left ventricle systolic depression; electrocardiogram anomalies, rhythm, mitral regurgitation, and coronary were also showed. Despite significant differences in clinical presentations and the need for intensive care at the admission, in all patients, cardiac involvement completely recovered after treatment. Cardiologic findings at the 6-month follow-up support the idea that cardiac involvement in MIS-C is a reversable and transient condition. In our cohort, all patients, even those with severe cardiac involvement in the acute phase, recovered their health completely in a relatively short time frame (6 months), and this supports the idea that cardiac dysfunction could be related to the hyper-inflammatory state that causes myocardial stunning, perivascular edema, and cytokine storm and—only in the minority—direct myocardial post-viral immunomediated damage. Additionally, the altered BNP plasma levels could suggest the role of the heart as an endocrine organ, participating in cardiovascular homeostasis and metabolism in addition to its pumping function in the acute phase of disease. This aspect could, however, explain the link between endo-metabolic derangement and heart involvement at admission.

Respiratory symptoms are not a prominent feature in MIS-C, but some children may present, e.g., cough, dyspnea, and in some case, the need for ventilation (68, 69).

No patients in our cohort needed invasive ventilation, in contrast with a recent metanalysis reporting a percentage of up to 30% of children needing mechanical ventilation (70).

However, it should be noted that in our cohort, the majority of patients were white, while in the metanalysis by Yasuhara et al., most of patients were not white, suggesting that race/ethnicity may affect the disease course and also the severity of organ involvement.

In contrast to adult COVID-19, primary respiratory failure does not seem to be a predominant cause for ICU admission in MISC.

Lung involvement has been hypothesized to be secondary due to cardiogenic pulmonary edema or primary due to the cytokine storm itself or to a direct parenchymal damage caused by the virus (69). In our population, at the MCA, no association between heart and lung involvement was noted, suggesting that they may be organ targets independent of the other. During the acute phase, while all patients had complete cardiac recovery after treatment, lung US was still altered in some patients before discharge, supporting that the lung needed a longer recovery time compared to other organs, despite less severe involvement.

At six months, some patients in our cohort referred to shortness of breath during exercise, in agreement with literature data (71), while lung US as well as echocardiogram were both unremarkable. Overall, these findings seem to support the hypothesis that lung involvement is probably mostly due to the cytokine storms of the acute phase, which do not provoke anatomical damage.

Children with MIS-C may present various neurological complications, some with a severe prognosis (9, 72, 73). In our sample, the incidence of neurological involvement was found to be much higher than the rates of up to around 50% reported in the literature (9, 72, 73); this difference is very likely due to the fact that the patients in the present study were all assessed, at admission and during the follow-up, by a child neurologist who was able to detect and evaluate even mild neurological signs and symptoms, and it confirms that specialist assessment of neurological aspects should be routinely included in the workup of patients with MIS-C. Although very frequent, the neurological involvement observed in our sample during the acute phase was not serious in most cases and the majority of the affected children had mild and short-lasting symptoms. This is in line with the findings of Larovere et al., who reported transient symptoms in 88% of their subjects with neurological involvement (73). In the most seriously affected children, the clinical picture was dominated by encephalopathy that disappeared with immunomodulatory therapy. Neurological assessment at discharge was normal, EEG improved from the first week, and in the majority of children, background activity normalized within two or three weeks.

At the six-month follow-up, the neurological assessment, again carried out by a child neurologist, focused not only on the clinical examination and EEG, but also on cognitive functions (non-verbal intelligence, *ad hoc* structured interview focusing on school performance, attention, and memory), behavioral and adaptive functioning, and quality of life. Severe neurological sequelae and EEG abnormalities were no longer present, but at this time, the majority of children presented cognitive difficulties (attention and memory disturbances), psychological difficulties (i.e., anxiety, depression), behavioral and adaptive problems (i.e., sleep disturbances or decline in academic performance), or low QoL.

Our observations suggest that the spectrum of neurological involvement in this syndrome could be wider and more complex than is currently thought. The neurological involvement during MIS-C tends to be dominated by the signs of acute encephalopathy that usually disappear, but follow-up allowed us to document cognitive, psychological, and behavioral sequelae in the majority of children. The severities of these sequelae are not related to the severities of systemic involvement but need to be addressed.

Gastrointestinal symptoms are recognized to be associated with the presentation of MIS-C (74). GI signs and symptoms and US alterations also appear prominently as presenting features of MIS-C in our patients, mimicking GI infection or even inflammatory bowel disease and surgical conditions. The GI tract is known to have high expression of ACE2 receptors in the human body, leading to viral entry into the cell. Additional mechanisms are proposed to explain the gastrointestinal involvement, such as indirect immune-mediated injury triggered by a systemic inflammatory response and disruption of the intestinal microecological balance, leading to excessive inflammation of the gut (74). During follow-up, complete intestinal recovery was detected, supporting that indirect injury, more so than cell damage, could be a crucial player in the GI symptoms that occur in MIS-C presentation.

Kidney involvement in children with MIS-C is common, as noted in our patients. Several reports described a direct viral tropism of COVID-19- and MIS-C-associated renal damage (75). Additionally, the abnormal immune response to the virus leading to tubular injury and podocytopathy, inflammatory process, hemodynamic instability, and vascular endothelial dysfunction has been described (76). Even though we only considered an increase in creatinine as a sign of poor kidney function, renal involvement seems to be limited at the acute phase of the disease, and long-term renal sequelae were not noted.

We recognize some limitations of the study. The small sample size limits the strength of the analysis; further multicenter studies are necessary to increase the sample size to extend and validate these results. We were unable to compare metabolic and endocrinological data of the same patients before and during MIS-C to define the direct influence of COVID-19 on marker levels; however, the anamnestic data can support the exclusion of a pre-existing disease. Finally, the neurological scores were defined differently at admission and follow-up times, including psychological difficulties and/or behavioral and adaptive problems at the 6-month evaluation; this aspect can limit the interpretation of the impact of COVID-19 on long-term neurological impairment.

## 5. Conclusions

MIS-C represents a critical illness in children and adolescents, requiring pediatric intensive-care unit (PICU) admission in most cases. Endocrinological and metabolic derangement; cardiac injury; respiratory, renal, and gastrointestinal manifestations; and neurological involvement are part of the initial presentation. The severity of initial clinical presentation may be associated with the organ domain; however, it is not related to the presence of long-term sequelae. During follow-up, multiorgan damage appears to resolve, except for the endocrine and neurological systems, which showed long-term abnormalities. Long-term follow-up allowed us to document cognitive, psychological, and behavioral sequelae in the vast majority of children. The prevalent organ restoration supports a predominant indirect immune-mediated injury triggered by a systemic inflammatory response; however, considering the long-term sequela, direct damage due to the viral entry could be not excluded. Even though our preliminary results seem to suggest that MIS-C is not a long-term risk condition for child health, a longer follow-up is necessary to confirm this hypothesis. Finally, long-term monitoring in larger samples is also needed to evaluate the infection impact on cognition and development.

Since MIS-C in children is a life-threatening event, vaccination strategies could be considered as preventive tools. The role of COVID-19 vaccine in prevention of severe infection among children, particularly in at high-risk children (77, 78), is reported and among healthy children the chances of MIS-C are more prevalent in the absence of vaccination (79–82). In patients with a history of MIS-C, an International consensus on vaccination against SARS-CoV-2 is hampered by a lack of evidence both on safety and efficacy (83).

## Data Availability

The original contributions presented in the study are included in the article/supplementary files, further inquiries can be directed to the corresponding author/s.
